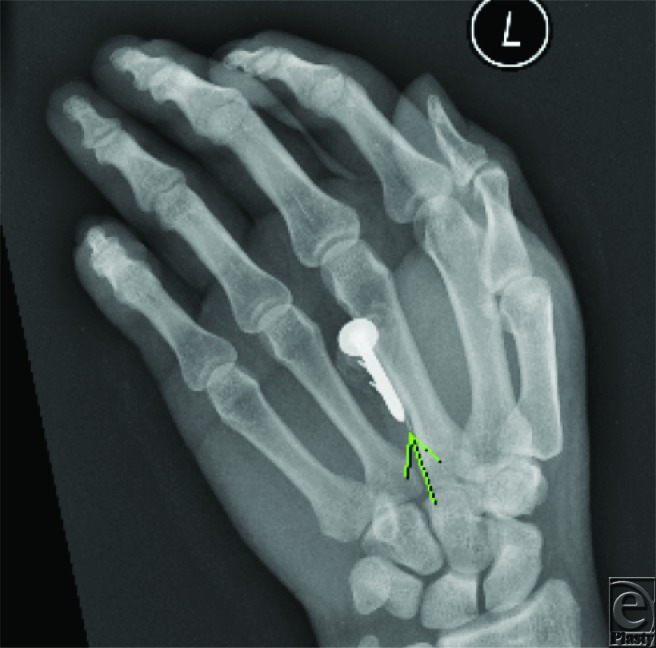# Barbed Screw Through the Hand

**Published:** 2012-04-12

**Authors:** Alexis Lanteri, Ruth Celestin, Earl Fleegler

**Affiliations:** Department of Surgery, Division of Plastic Surgery, University of Medicine and Dentistry of New Jersey—New Jersey Medical School, Newark, NJ

## DESCRIPTION

A 25-year-old right-hand dominant man presents with an accidental self-inflicted nail gun injury impaling the mid left palm in line with the middle finger and close to Kaplan's line.

## QUESTIONS

1. **Describe mechanisms of nail-gun injury.**
2. **Discuss initial workup including history and information about physical examination.**
3. **What findings on physical examination are indicators for surgical exploration?**
4. **Describe the specifics of surgical exploration in this patient.**

## DISCUSSION

Nail guns are commonly used construction tools powered by a high-velocity explosive charge or low-velocity compressed air. Most nail gun injuries occur during routine use and are due to accidental discharge from careless handling or structural unsoundness of the receiving material. Typically the radial aspect of the nondominant hand is most commonly involved as it is used to grip or steady the structure being nailed.

Hand injuries can result in bone, joint, tendon, nerve, and/or soft-tissue injury. The majority of nail-gun injuries are isolated soft tissue injuries. When penetrating tissue, the nail's kinetic energy forms expanding shock waves, which crush nearby structures. Injuries may be further complicated by contamination with skin bacteria, oil, paper, or glue. Barbs, or copper wire fragments imbedded in the nail may become lodged within the tissue and require complicated extraction.

A careful history should be taken when evaluating this injury, with attention to the type of nail gun and the tetanus immunization status of the patient. Physical examination should first note the general appearance of the hand and the proximity of the laceration to important anatomic structures. Any deficit in digit range of motion suggests injury to either one of the tendons within the hand or to the median or ulnar motor nerves. In addition, assessing capillary refill provides information about injury to the palmar arch, and examining sensation to light touch allows evaluation of sensory nerve injury. Radiographs should also be analyzed to determine joint involvement, fractures, and presence of barbs.

In treating this injury, antibiotics should be administered and the wound should be cleaned, explored, and debrided under a regional block. If the patient presents with absent pulses, an insensate digit, suspected joint penetration, fracture, or tendon injury, immediate surgical consultation and open exploration should be performed.

In the aforementioned case, open exploration and extraction of a barbed nail under direct visualization was necessary to avoid secondary damage. The palmar aponeurosis was incised and sensory nerves were identified and protected. The transverse carpal ligament was identified and divided into the deep forearm fascia. The median nerve was found to be compressed in the mid carpal tunnel area due to traumatic edema. The nail was located distal to the superficial vascular arch and beside the common digital artery. Two barb like fragments extended through the overlying lumbrical muscle and flexor tendons into the middle finger. These muscle and tendons were elevated off the barbs and the nail was removed. The hand was irrigated with sterile normal saline, debrided, and sutured.

## Figures and Tables

**Figure:**